# Adverse clinical events caused by pacemaker battery depletion: two case reports

**DOI:** 10.1186/s12872-020-01622-x

**Published:** 2020-07-23

**Authors:** Junpeng Liu, Li Wen, Simin Yao, Peipei Zheng, Shuang Zhao, Jiefu Yang

**Affiliations:** 1 Department of Cardiology, Peking University Fifth School of Clinical Medicine, Beijing Hospital, National Center of Gerontology, Institute of Geriatric Medicine, Chinese Academy of Medical Sciences, No.1 Da Hua Road, Dong Dan, Beijing, 100730 P.R. China; 2 Department of Emergency Medicine, Peking University Fifth School of Clinical Medcine, Beijing Hospital, National Center of Gerontology, Institute of Geriatric Medicine, Chinese Academy of Medical Sciences, No.1 Da Hua Road, Dong Dan, Beijing, 100730 P.R. China; 3 State Key Laboratory of Cardiovascular Disease, Arrhythmia Center, Fuwai Hospital, National Center for Cardiovascular Diseases, Chinese Academy of Medical Sciences and Peking Union Medical College, Beijing, 100037, P.R. China

**Keywords:** Pacemaker battery depletion, Pacemaker syndrome, Atrioventricular block-induced torsade de pointes, Electrocardiogram

## Abstract

**Background:**

The clinical symptoms and adverse events caused by pacemaker battery depletion are not uncommon, but it is easy to miss or misdiagnose them clinically. To raise the level of awareness towards this clinical situation, we report two cases.

**Case presentation:**

We described two cases of pacemaker battery depletion. Case 1 was an 83-year-old male manifesting chest pain and dyspnea. Automatic reprogramming after pacemaker battery depletion resulted in pacemaker syndrome. While case 2 was an 80-year-old female with complete atrioventricular heart block and torsade de pointes, due to complete depletion of pacemaker battery. In addition, we introduce a method that can easily identify the depletion of the pacemaker battery, which has clinical promotion value of a certain degree.

**Conclusions:**

Those cases emphasize that serious morbidity can arise from pacemaker battery depletion, even in the early stages. Therefore, early detection and diagnosis is especially important.

## Learning points

Chest pain, dyspnea accompanied by changes of pacing mode and rate in patient with pacemaker suggest the possibility of battery depletion of pacemaker.

The symptoms resolution following pacemaker exchange support this theory.

Torsade de pointes(TdP) tachycardia in patient with pacemaker suggests the possibility of pacemaker battery depletion, which causes bradycardia with QT interval prolongation.

## Background

Pacemaker battery depletion is a gradual process, usually divided into two stages, ERI (Elective replacement indication) and EOL (End of life) [1–3]. First stages is the ERI status, usually lasting for 3–6 months. At this stage, in order to extend battery life, the pacemaker will automatically reprogram, including turning off the rate response function, and changing in pacing mode and rate. If the generator is not replaced during this period, it will progress to EOL status and the pacemaker will gradually stop working, causing varying degrees of symptoms and clinical events [4–6]. Meanwhile, due to lack of understanding of and attention to it, it is easy to be misdiagnosed and missed diagnosis in the Emergency Department(ED).

We present two cases related to pacemaker battery depletion in order to improve the understanding of this special clinical situation.

## Case presentation

### Case 1

An 83-year-old male was admitted to our ED due to angina pectoris and dyspnea. He had a past medical history of coronary heart disease and hypertension. He received a pacemaker due to sick sinus syndrome. A new generator (DDDR, ADDR01, Medtronic) was replaced because of the pacemaker battery depletion 8 years ago. He had not followed up with the pacemaker in the past 3 years.

**Fig. 1:**
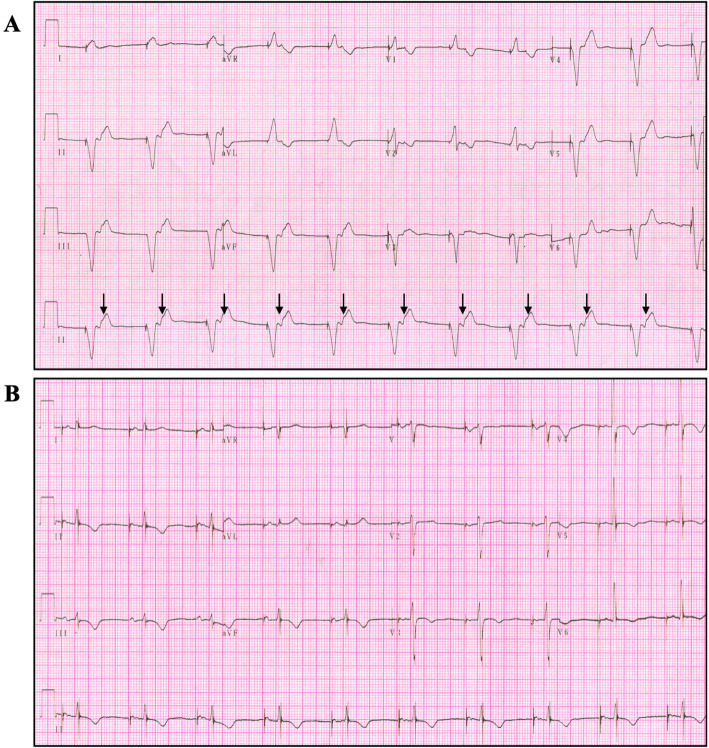
ECG (**a**: at presentation **b**: after pacemaker exchange). **a** Non-synchronous ventricular pacing at a fixed of 65 bpm with retrograded atrial capture (black arrow). **b** Atrial pacing following by a spontaneous ventricular rhythm

In ED, the patient was awake, his blood pressure was 146/79 mmHg, heart rate 65 bpm with regular rhythm, and lower limbs mild edema. Laboratory studies reported: troponin T 0.01 ng/ml(< 0.014 ng/ml), CK-MB 1.77 ng/dl(< 4.99 ng/dl) and N-terminal pro brain natriuretic peptide (NT-proBNP) 2458 pg/ml(< 125 pg/ml). The electrocardiogram (ECG) demonstrated right ventricular pacing at a rate of 65 beats/min, and retrograde P waves can be seen, indicating atrioventricular dyssynchrony. The echocardiography showed tricuspid moderate regurgitation, aortic valve calcification with mild insufficiency, left ventricular ejection fraction(LVEF) was 50%. Treatments such as anti-angina and diuretic therapy did not ease the symptoms. Soon after, pacemaker interrogation was performed with automatic reprogramming was triggered by an alert for ERI, with changes in the pacing mode (from DDDR to VVI) and the pacing rate, which was fixed at 65 bpm. Therefore, we concluded that the patient’s symptoms corresponded to pacemaker syndrome, which included absence of rate response to physiological need, loss of atrioventricular synchrony and retrograde ventriculoatrial conduction. After the replacement of the generator, ECG showed atrial pacing at a rate of 62 bpm followed by a spontaneous ventricular rhythm (Fig. 1b), and at the same time, the symptoms of the patients were immediately disappeared, NT-proBNP decreased to 347 pg/ml.

### Case 2

An 80-year-old woman was admitted to our ED with fever and syncope. The patient received a pacemaker (DDDR, Cylos DR, Biotronik) due to third-degree atrioventricular(AV) block 9 years ago. She was bedridden because of stroke sequelae and loss of pacemaker follow-up for the last 2 years. She had a history of hypertension and was prescribed aspirin, amlodipine as regular oral medications.

**Fig. 2:**
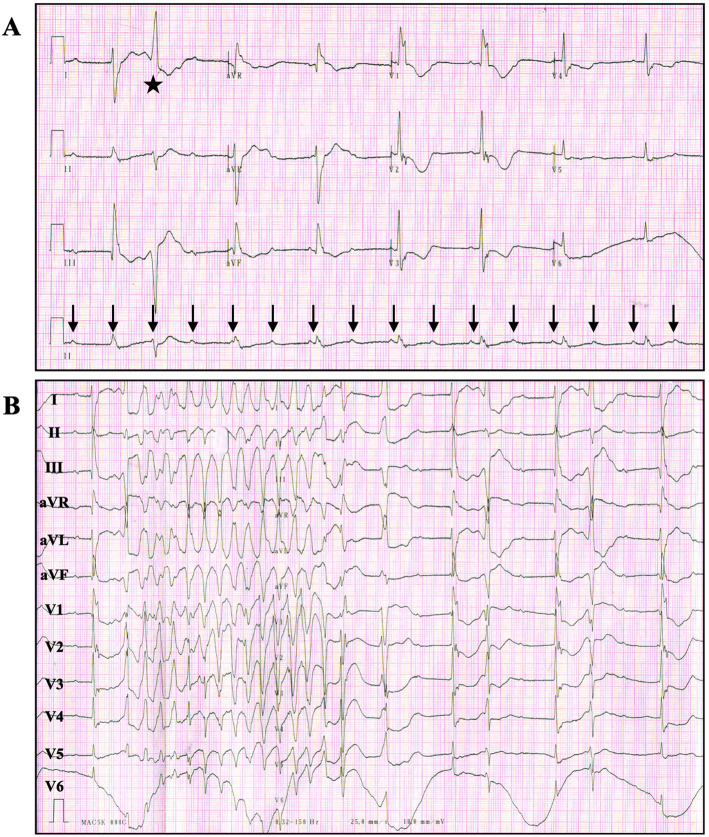
ECG (**a** and **b**: at presentation). **a**: Completely dissociated P waves (black arrows) and wide QRS complexes. The rhythm strip showed complete atrioventricular(AV) block. The QRS complexes were wide because of the presence of right bundle branch block and left posterior fascicular block, representing the AV block is infranodal. QT intervals were prolonged (QTc 550 ms) and premature ventricular contraction(PVC) was present (black star). **b**: Complete AV block with wide QRS complexes, frequent PVCs were present. The first PVC occurred on the downslope of the T wave, which induced a polymorphic ventricular tachycardia with changing QRS complex amplitudes, which was known as torsade de pointes(TdP)

On arrival, the patient was generalized weakness. Physical examination revealed blood pressure of 86/40 mmHg, the body temperature was 38 degree Celsius. During monitorization and ECG examination (Fig. 2), we observed third-degree AV block with QT interval prolongation, and the premature ventricular contraction(PVC) was accompanied by torsade de pointes(TdP). Laboratory analysis showed blood potassium 5.0 mmol/L and blood calcium 2.19 mmol/L. Cardiac biomarkers, liver and renal function were normal. An isoproterenol infusion and intravenous magnesium sulfate(MgSO4) treatment was initiated. In order to find out the reason why the pacemaker was not working, pacemaker interrogation was performed immediately. However, the pacing system programmer could not interrogate the device, and the magnet check did not respond, indicating pacemaker battery depletion in the EOL status. During the preparation for temporary pacing, ventricular fibrillation occurred and she died after rescue.

## Discussion and conclusions

The clinical effects of pacemaker battery depletion come from two aspects: 1. Automatic reprogramming of the pacemaker: It occurs in the early stage of battery depletion (ERI period), and it has adverse clinical repercussions in some patients related to loss of rate response, loss of atrioventricular synchrony and ventricular synchrony, changes in lower rate limit (Usually decreases by 10 to 11%, or becomes a fixed rate), or a combination of these factors [3, 5]. Sinha et al. [5] surveyed the cardiorespiratory symptoms and adverse clinical events of 266 patients with pacemaker battery depletion in the ERI status. The results showed that 83 patients (31.2%) had symptoms and 28 patients (10.5%) had clinical events, associated with clinical conditions included heart failure (32%), chest infection (21%), pacemaker syndrome (18%), pre-syncope (14%), and palpitations (11%). 2. Bradycardia: With the progress of pacemaker battery depletion, the symptoms will gradually worsen. Syncope and pre-syncope were reported to be the most frequent symptoms [4, 6]. However, syncope in bradycardia is not always caused by asystole but may instead be caused by TdP. TdP is a fatal polymorphic ventricular tachycardia, which is caused by many conditions that prolonged QT interval, such as drug, electrolyte abnormalities, bradycardia, myocardial infarction, congestive heart failure et al. [7]. In Case 2, no electrolyte abnormalities were found and she was not taking drugs that were known to prolong the QT interval. Cardiac biomarkers were negative and the evidence for the diagnosis of myocardial infarction was insufficient. Although complicated with heart failure and fever, it could not fully explain the QT interval prolongation. Complete atrioventricular block was the main cause of prolongation of QT interval [8].When associated with premature ventricular contractions, it was very easy to induce TdP. However, the predictors of TdP in AV block-induced acquired long QT syndrome(LQTS) are not well defined. Previous studies suggested that female sex, the increased time from the peak to the end of the T wave, a combination of LQT2-like notched T waves, or cardiac memory resulting from a change in QRS morphology [9–11]. Therefore, syncope in AV block may be caused by malignant ventricular tachycardia. When patients with pacemaker have unexplained ventricular arrhythmias, especially TdP, we should be alert to the possibility of pacemaker battery depletion, and the placement of a temporary pacer could be the key to treatment.

Timely diagnosis of pacemaker battery depletion is crucial, helping to take targeted treatment. However, many health care providers do not have ready access to pacemaker interrogation. Carison et al. [12] presented a simple method to predict whether the pacemaker has progressed the ERI status through the ECG alone, that is, the “Rules of ten”, and the patient can be diagnosed by meeting any of the following:1. Atrial pacing not at a multiple of ten; 2. Non-synchronous ventricular pacing not at a multiple of ten. The diagnostic criteria have a sensitivity of 79% and a specificity of 92.6%. It should be noted that this method is relevant to 3 major pacemaker manufactures (Abbott, Biotronik, Medtronic) excerpt Boston Scientific and MicroPort. This is because most manufacturers decrease the lower rate limit when reprogramming occurs. When Abbot’s, Biotronik’s, and Medtronic’s pacemakers are automatically reprogrammed, the lower rate decreased by 10,11%, and to 65 bpm, respectively (Table 1). The ECG performance of case 1 complies with the second diagnostic criteria. The non-synchronous ventricular paced at a fixed rate of 65 bpm, which violates a multiple of ten, so the pacemaker battery could be diagnosed as exhausted.

**Table 1:** Manufacturer-Specific Pacemaker Programming at Replacement Notification

Pacing characteristic	Boston Scientific	Abbott	Biotronik	Meditronic	Microport
Loss of Rare Response?	Yes	Yes	Yes	Yes	Yes
Loss of AV Synchrony?	No	No	No	Yes(VVI)	Yes(VVI)
Change in Lower Rate Limit?	No	Yes (−10%)	Yes (−11%)	Yes (65 bpm)	Yes (70 bpm)
Magnet rate following replacement Notification	≤85 bpm	≤86.3 bpm	≤80 bpm	≤65 bpm	≤80 bpm

In conclusion, these cases emphasize that serious morbidity can arise from pacemaker battery depletion, even in the early stages. We not only need to improve the understanding of how to detect clinically pacemaker battery depletion, but also should pay attention to the follow-up of pacemaker patients.

## Supplementary information

**Additional file 1.**

## Data Availability

Not applicable.

## Abbreviations

AV: Atrioventricular
ECG: Electrocardiography
ED: Emergency department
EOL: End of life
ERI: Elective replacement indication
LQTS: Long QT syndrome
LVEF: Left ventricular ejection fraction
NT-proBNP: N-terminal pro brain natriuretic peptide
PVC: Premature ventricular contraction
TdP: Torsade de pointes
