# Supplementary material for: Adverse clinical events caused by pacemaker battery depletion: two case reports
Source: BMC Cardiovasc Disord. 2020 Jul 23;20:344. doi: 10.1186/s12872-020-01622-x (PMC7376836; doi:10.1186/s12872-020-01622-x)
Supplement: Supplementary file 1 — Additional file 1. [file 12872_2020_1622_MOESM1_ESM.docx]

Timeline of case 1

| Events | |
| --- | --- |
| Initial presentation | Angina and dyspnea |
| At the emergency room | Electrocardiogram with ventricular pacing at 65 bpm |
|  | NT-proBNP 2458pg/ml, troponin T 0.01ng/ml |
|  | Echocardiography with tricuspid moderate regurgitation, aortic valve calcification with mild insufficiency, left ventricular ejection fraction is 50% |
|  | Anti-angina and diuretic therapy |
| 24h post-arrival  After 2 days | Symptoms were not relieved  Pacemaker interrogation with ERI  Pacemaker exchange  Electrocardiogram with atrial pacing followed by a spontaneous ventricular rhythm |
|  | Symptoms resolution  NT-proBNP 347pg/ml |

Timeline of case 2

| Events | |
| --- | --- |
| Initial presentation | Syncope and fever |
| At the emergency room | Blood pressure 86/40mmHg |
|  | Electrocardiogram with third-degree atrioventricular block with QT interval prolongation, and ventricular premature beat was accompanied by torsade de pointes |
|  | Blood potassium 5.0mmol/L, calcium 2.19mmol/L |
|  | Cardiac biomarkers were normal  Isoproterenol and magnesium sulfate infusion |
| 1h post-arrival | Pacemaker interrogation with EOL  Temporary pacing to prepare  CPR was initiated, there wasn’t return of spontaneous circulation |
| 2h post-arrival | Patient decease |
